# Editorial: Motor recovery following central neurological disorders in humans: Mechanisms and therapeutic interventions

**DOI:** 10.3389/fnhum.2023.1171193

**Published:** 2023-04-03

**Authors:** Silvi Frenkel-Toledo, Jason Friedman, Dario G. Liebermann, Yaron Sacher, Patrice L. Weiss

**Affiliations:** ^1^Department of Physical Therapy, School of Health Sciences, Ariel University, Ariel, Israel; ^2^Department of Neurological Rehabilitation, Loewenstein Rehabilitation Medical Center, Ra'anana, Israel; ^3^Department of Physical Therapy, Sackler Faculty of Medicine, Stanley Steyer School of Health Professions, Tel Aviv University, Tel Aviv-Yafo, Israel; ^4^Sagol School of Neuroscience, Tel Aviv University, Tel Aviv-Yafo, Israel; ^5^Department of Rehabilitation, Ministry of Health, Jerusalem, Israel; ^6^ALYN Hospital Children's and Adolescent Rehabilitation Center, Jerusalem, Israel; ^7^Department of Occupational Therapy, University of Haifa, Haifa, Israel

**Keywords:** neurological disorders, neurorehabilitation, neuroplasticity, mechanism, therapy, rehabilitation technology, motor recovery

Central neurological disorders (e.g., stroke and traumatic brain injury) are a leading cause of acquired long-term motor disability. For many people with such disorders, independence in daily activities and quality of life remain impaired. Rehabilitation aims to facilitate and enhance the recovery of motor function using a combination of restitution-oriented and/or compensation-oriented treatment strategies. Restitution refers to restoring movement quality, while compensation refers to learning new ways to use the residual capacity to accomplish a task. Yet, rehabilitation training designed to improve motor recovery has partially succeeded only in experimental trials.

Neural reorganization is a critical driver of motor recovery. Still, it can also produce maladaptive effects and may eventually interfere with motor recovery in some people that suffer from central neurological disorders. Despite some advances in the understanding of mechanisms of motor recovery, current knowledge about its underlying (patho-) physiological processes remains scarce. Therefore, it is important to understand the factors that enhance or prevent motor recovery and develop informed therapeutic interventions based on neurobiological knowledge and evidence.

This special issue includes seven articles to bring together relevant scientific domains that highlight the interdisciplinary approach common to studies on these topics. They address both theoretical and practical issues.

Three out of the seven papers are focused primarily on elucidating mechanisms underlying motor ability. In *Mixed Neuropathologies, Neural Motor Resilience and Target Discovery for Therapies of Late-Life Motor Impairment*, Buchman and Bennett propose that investigators can identify genes and proteins related to neural motor resilience. They isolate motor decline due to aging from neuropathology and degeneration and aim to enhance the understanding of the heterogeneity in late-life motor impairment by identifying molecular mechanisms. A second achievement is related to revealing therapeutic targets that have high value for the discovery of drug therapies that could help to relieve often untreatable motor impairments.

In *Different patterns of neural activity characterize motor skill performance during acquisition and retention*, Beroukhim-Kay et al. examine whether brain activity in the primary motor cortex and basal ganglia of 40 neuro-typical young adults during practice is related to trial-by-trial practice performance and to determine whether it is predictive of immediate recall performance. They used whole-brain analysis of functional magnetic resonance imaging and behavioral performance measures. Their findings demonstrate that improved practice performance and recall of a sensorimotor skill are correlated with distinct neural activity patterns during acquisition, drawing on different motor learning mechanisms during encoding. They suggest that the different neural mechanisms engaged in motor learning and performance may inform novel interventions to enhance motor skill learning.

In the article, *Developmental and acquired brain injury have opposite effects on finger coordination in children*, Mimouni-Bloch et al., compare finger coordination in a group of children aged 4–12 with cerebral palsy (CP) and TBI to a group of typically developing children using an isometric pressing task. Deficits were observed in a test of hand function for both pathological groups, and children in both groups performed the pressing task worse than the control group. However, while children with CP improved their finger coordination as they matured, children with TBI showed the opposite pattern, with worse finger coordination in older children. Between-group differences may be a result of different areas of brain injury typically observed in CP and TBI, and the different amount of time that has passed since the injury.

The other four papers delve into identifying effective theory-driven therapeutic interventions and technology-based solutions for improving motor recovery. Munoz-Novoa et al. present the article *Upper limb stroke rehabilitation using surface electromyography: A systematic review and meta-analysis*. Overall, the evidence does not support an effect of surface electromyography (sEMG) compared to a non-sEMG intervention, nor does it establish that an sEMG intervention is the most effective treatment for improving upper limb function in stroke populations. Nevertheless, the authors establish that sEMG is a promising tool to improve functional recovery further. They conclude that randomized clinical trials with larger sample sizes are needed.

In *Retrospective Analysis of Task-Specific Effects on Brain Activity After Stroke: A Pilot Study*, Demers et al. evaluate functional brain activation changes during a precision and a power grasp task in eight chronic stroke survivors following a 2-week training session of constraint-induced movement therapy (CIMT), compared to six patients in a no-treatment control group. CIMT resulted in a relative increase in activity in the dorsal premotor cortex of the lesioned hemisphere under precision grasp task conditions compared to the non-treatment control group. The results point to initial evidence for task-specific effects of CIMT, thereby supporting the use of CIMT to enhance the recovery-supportive cortical reorganization for this population.

In the article *Automating provision of feedback to stroke patients with and without information on compensatory movements: A pilot study*, Fruchter et al. test the ability of a rule-based set of guidelines to determine the optimal hierarchy, timing, and content of feedback to be used when stroke patients train with an upper-limb exercise on 11 stroke patients, 1–72 months from injury onset. Participants preferred the configuration that provided feedback on task success and movement quality.

Avraham et al. present an article titled *Skill-learning by observation-training with patients after traumatic brain injury*, where they used observational training to examine the acquisition of procedural memory in patients with Traumatic Brain Injury (TBI). They tested improvement one day and 2-weeks after training. The results demonstrated the importance of procedural memory consolidation and retention through observational learning for TBI patients. In addition, different functional traits may predict the outcomes of observational training.

To conclude, this collection of articles illustrates the important clinical insights made by current research trends examining mechanisms underlying motor ability and recovery and rehabilitation training to enhance motor recovery. Future research should continue to focus on developing new therapeutic interventions based on neurobiological knowledge, while taking into consideration traits that can identify the most responsive patients toward a specific therapeutic intervention.

## Author contributions

PW wrote the first draft of the manuscript. All authors contributed to manuscript revision, read, and approved the submitted version.

